# Supralevator abscess: New treatment for an uncommon aetiology: Case report

**DOI:** 10.1016/j.ijscr.2019.05.016

**Published:** 2019-05-13

**Authors:** David João Aparício, Carlos Leichsenring, Cisaltina Sobrinho, Nuno Pignatelli, Vasco Geraldes, Vítor Nunes

**Affiliations:** Surgery, Hospital Professor Doutor Fernando Fonseca, IC 19, Lisbon 2720-276, Portugal

**Keywords:** Supralevantor abscess, Mechanic fistulae tract marsupialization, Case report

## Abstract

**Introduction:**

Supralevator abscess is the least common type of anorectal abscess. Its diagnosis can be hard and treatment difficult.

**Presentation of the case:**

A 48-year-old men was diagnosed in the emergency department with a supralevantor abscess. Under general anaesthesia, the abscess drainage was accomplished after removal of a fish bone, who was perforating the rectum. Due to persistent rectal purulent discharge, a pelvic Magnetic Resonance (MRI) was performed: a supralevator abscess adjacent to the internal obturator muscle and an inter-sphincteric fistulae from the inferior margin of this collection were identified. A Pezzer® drain was placed through the fistula tract. After radiological resolution, under general anaesthesia, the patient was submitted to extraction of the drain and marsupialization of the path left using an ENDO GIA®. At two year follow up he remained asymptomatic.

**Discussion:**

Despite of the abscess aetiology, the principles of treatment are the same: good radiological characterization and proper drainage. An adequate radiological characterization is important to avoid iatrogenic creation of a complex fistulae.

**Conclusion:**

If a supralevator abscess diagnosis is made, fistulae trajectory should be studied. If no clear internal opening is evident, a pelvic MRI should be done followed by drainage of the abscess. After resolution the drain should be taken off and marsupialization with ENDO GIA® should be performed.

## Highlights

• Proper drainage of supralevantor abscess should be achieved for the fistulae path.
• After supralevator abscess resolution the drain should be taken off and marsupialization with ENDO GIA® should be performed.
• It is possible to adapt the length of ENDO GIA® to the length of the fistulae tract.
• This treatment is a safe method for definitive treatment of traumatic supralevator abscess with intersphincteric fistulae.

## 1 Introduction

Anorectal abscess is a common surgical problem in the emergency care. The original infection is cryptoglandular in 90% of cases. Other aetiologies result from downward spread of pelvic infection, inflammatory bowel disease or traumatic incidents [1].

Supralevator abscess is the least common type of anorectal abscess (less than 10%) [2,3]. Its diagnosis can be challenging because external signs may be absent. Treatment may be difficult with incomplete drainage frequently occurring, which can justify higher recurrence rates, as well as re-interventions and potentially life-threatening complications [2].

This work was reported in line with the SCARE criteria [11].

## 2 Presentation of case

A 48-year-old caucasian men was admitted to the emergency department for localized pain in the hypogastrium and left iliac fossa, fever, absence of passage of feces and anal pain for four days evolution. The physical examination revealed pain in the hypogastrium and left iliac fossa; digital rectal examination was not possible due to anal pain.

**Fig. 1:**
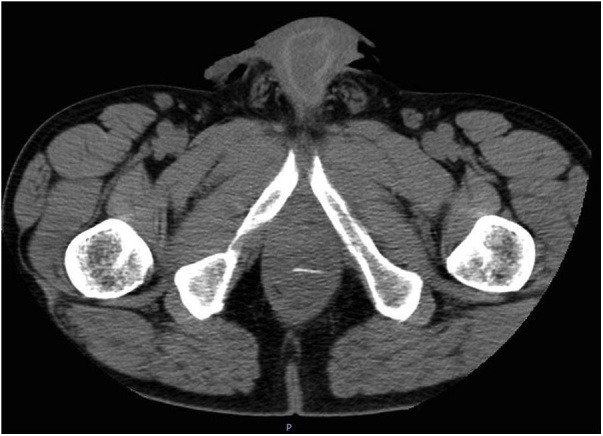
Pelvic computerized tomography (CT) revealed a sharp radiopaque image on the rectum.

**Fig. 2:**
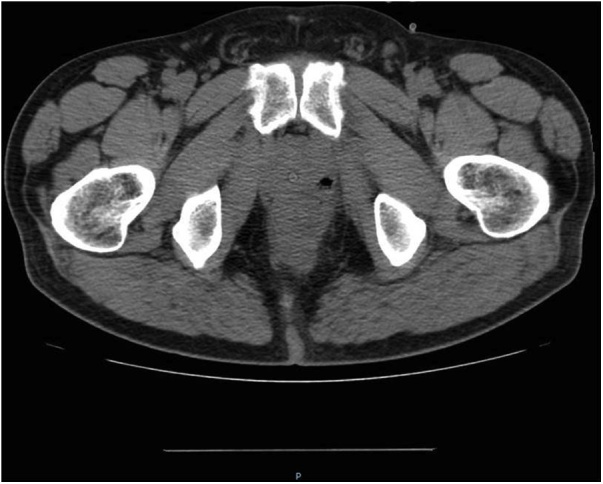
Pelvic computerized tomography revealed a left perianal heterogeneous hypodense collection with 36 × 12 mm.

Pelvic computerized tomography (CT) revealed a sharp radiopaque image on the rectum; a left perianal abscess with 36 x 12 mm and densification of perirectal adipose tissue (Figs. 1,2).

**Fig. 3:**
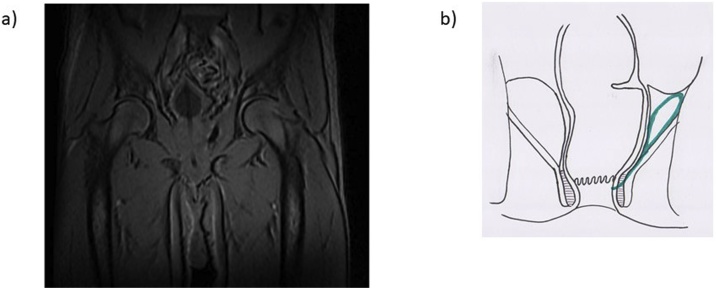
a)Pelvic nuclear magnetic resonance revealed a 6 × 2 × 3 cm supralevator abscess with one major inter-sphincteric fistulae from the inferior margin of this collection; b) illustration of supralevantor abscess (green).

The patient started antibiotherapy and was proposed to abscess drainage under general anaesthesia. After being positioned in a lithotomy position, a 3 cm fish bone perforating the rectum, adjacent to a supralevantor abscess, at 9 cm from the anal margin was identified. After fish bone removal, abscess drainage and lavage were accomplished. On the sixth post-operative day due to persistent rectal purulent discharge, a pelvic MRI was performed and a 6 × 2 x 3 cm supralevator abscess adjacent to the prostatic lateral face and to the internal obturator muscle, one major inter-sphincteric fistulae from the inferior margin of this collection, with an inferior and posterior direction, with a length of 2 cm were identified (Fig. 3).

**Fig. 4:**
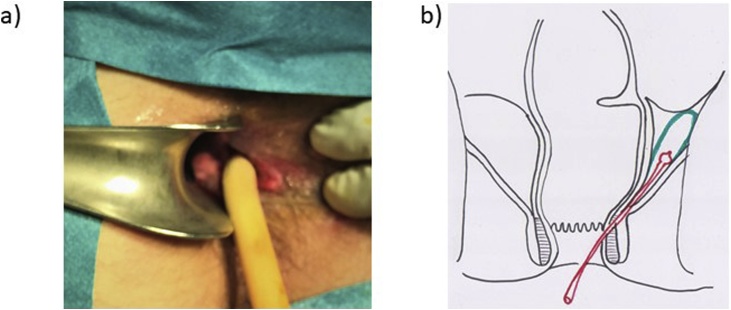
a) Pezzer® (P0) drain through the fistula tract; b) illustration of the supralevantor abscess (green) with the drain (red) trough inter-sphincteric fistulae.

**Fig. 5:**
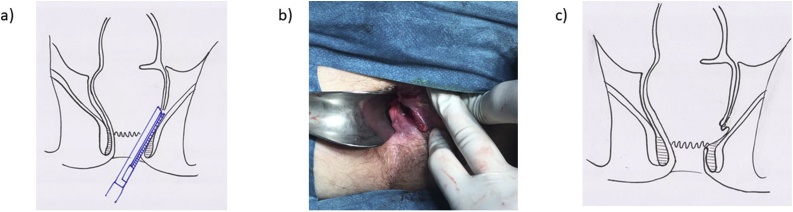
a) Illustration of the marsupialization of the path left by the drain (3 cm) using a ENDO GIA^®^ 30 - 3.5 mm cartridge (blue); b) final result after marsupialization; illustration of the final result.

The patient was submitted to a new drainage procedure under general anesthesia. In the lithotomy position, the inter-sphincteric fistula was identified, reaching the supralevator abscess. Drainage, washout, and placement of a Pezzer® (P0) drain through the fistula tract was done (Fig. 4). Twelve days after this surgery, during which daily washings of the abscess cavity were performed, the patient improved clinical and analytically, with radiological resolution of the abscess. He was proposed for definitive treatment, with extraction of the drain and marsupialization of the path left by the drain (3 cm) using a ENDO GIA^®^ 30 - 3.5 mm cartridge (Fig. 5).

The patient was discharged asymptomatic 4 days after definitive treatment. At two year follow up, he remained asymptomatic without recurrence.

## 3 Discussion

The majority of supralevator abscess originate from the glandular crypts [1,4]. In this case, it seems the abscess developed from traumatic perforation by a fish bone, spreading for an inter-sphincteric fistulae. Despite of the aetiology, the principles of treatment are the same: good radiological characterization and proper drainage. An adequate radiological characterization is important to avoid iatrogenic creation of a complex fistulae. Therefore, if an inter-sphincteric fistulae is present, the approach should be endorectal, in order to avoid supra-sphincteric fistulae [5,6]. Radiologic characterization may be achieved by pelvic MRI, endorectal ultrasound or pelvic CT, with the first two methods being considered the gold-standard [7]. Pelvic MRI is a good method to evaluate the disease extension and fistulae direction [7]. It modifies the surgical approach in 10% of the cases [8] and contributes to reduce the recurrence in 75% [8]. Endorectal ultrasound is better than MRI in identifying peri-rectal abscesses, complex fistulae and their internal opening. It can also assist abscess drainage [7]. Meanwhile, the criteria to perform either endorectal ultrasound or MRI in the emergency department are still not defined [7].

Treatment of a supralevator abscess is complex [6,9]. The patient should undoubtedly undergo drainage; however, if abscess anatomy is not clearly defined, it should not be done in an aggressive way and may be re-scheduled until a proper radiological study is available [9]. Subsequently, an adequate drainage should be executed and a Pezzer™ drain placed in the abscess space. If there’s an inter-sphincteric fistulae, the drain should be placed through the fistula’s opening or, if the opening is not evident, at the nearest point of the dentate line. The drain provides multiple advantages: it helps in abscess cavity collapse; allows abscess cavity washing; permits MRI imaging to monitor the abscess evolution; allows visualization of the path for an eventual definitive treatment, while the patient can live his normal daily life [9]. Drainage duration is variable and should be based on clinical, analytical, and radiological data [2]. In our case, on the first surgery, the abscess anatomy and the internal fistulae drainage opening was not clear, so we preferred to extract the fish bone and drain the abscess conservatively. After clinical and radiological worsening (pelvic MRI), a Pezzer drain® was placed in the abscess cavity through the inter-sphincteric fistulae.

Definitive surgical treatment in a second surgical time is still controversial [6,9]. A series published supported the absence of recurrence or incontinence when definitive treatment is performed through fistulae marsupialization [6,10]. Flap advancement after abscess drainage is not indicated, because primary failure of the flap is the expected outcome [10]. Conversely, when marsupialization is not executed, there is 50% risk of recurrence. Fistulae marsupialization could be performed with fistulae cut with or without energy and ensuing suture after or with a mechanical suture. Trans-anal suture may be a complex procedure and the use of ENDO GIA® provides less haemorrhage, less surgical time, avoidance of external sphincter lesions. Nevertheless, there is need of a minimal length of the fistulae tract to place the EndoGIA. To our knowledge, only 3 cases of this method were reported in the literature (2 from cryptoglandular and 1 from Crohn’s disease aetiology) using a ENDO GIA® 45 [10].

## 4 Conclusion

After supralevator abscess resolution and cavity collapse the drain should be taken off and marsupialization with ENDO GIA® should be performed. It is possible to adapt the length of ENDO GIA® to the length of the fistulae tract. This treatment is a safe and easy method for definitive treatment of traumatic supralevator abscess with intersphincteric fistulae.

## Conflicts of interest

All authors had not any financial and personal relationships with other people or organisations that could inappropriately influence their work.

## Sources of funding

No funding or sponsors for this article.

## Consent

Patient gave his consent for publishing this article.

## Ethical approval

This article was exempt from our Hospital ethnical committee, since is a case report and the patient gave his contempt to publish.

## Author contribution

David Aparício was envolved at study concept, data collection, writing the paper.

Carlos Leichsenring was envolved at study concept, data collection, writing and review the paper.

Cisaltina Sobrinho was envolved at study concept, data collection, writing and review the paper.

Nuno Pignatelli was envolved at study concept, data collection, writing and review the paper.

Vasco Geraldes was envolved at study concept, data collection, writing and review the paper.

Vitor Nunes was envolved at study concept, data collection, writing and review the paper.

## Registration of research studies

None.

## Guarantor

The Guarantor is the corresponding author (David Aparício).

## Provenance and peer review

Not commissioned, externally peer-reviewed.
